# Recurrence risk stratification of hepatocellular carcinomas based on immune gene expression and features extracted from pathological images

**DOI:** 10.1371/journal.pcbi.1011716

**Published:** 2023-12-29

**Authors:** Tao Ding, Xiao Li, Jiu Mo, Gregory Alexander, Jialu Li

**Affiliations:** 1 Department of Statistical Science, University College London, London, United Kingdom; 2 Product Development Personalized Healthcare, Genentech, San Francisco, California, United States of America; 3 Department of Computer Science, Central South University of Forestry and Technology, Changsha, Hunan, People’s Republic of China; 4 Mathematical Statistician Consultant, San Francisco, California, United States of America; Indiana University School of Medicine, UNITED STATES

## Abstract

**Background:**

Immune-based therapy is a promising type of treatment for hepatocellular carcinoma (HCC) but has only been partially successful due to the high heterogeneity in HCC tumor. The differences in the degree of tumor cell progression and in the activity of tumor immune microenvironment could lead to varied clinical outcome. Accurate subgrouping for recurrence risk is an approach to address the issue of such heterogeneity. It remains under investigation as whether integrating quantitative whole slide image (WSI) features with the expression profile of immune marker genes can improve the risk stratification, and whether clinical outcome prediction can assist in understanding molecular biology that drives the outcome.

**Methods:**

We included a total of 231 patients from the Cancer Genome Atlas Liver Hepatocellular Carcinoma (TCGA-LIHC) project. For each patient, we extracted 18 statistical metrics corresponding to a global region of interest and 135 features regarding nucleus shape from WSI. A risk score was developed using these image features with high-dimensional survival modeling. We also introduced into the model the expression profile of 66 representative marker genes relevant to currently available immunotherapies. We stratified all patients into higher and lower-risk subgroup based on the final risk score selected from multiple models generated, and further investigated underlying molecular mechanisms associated with the risk stratification.

**Results:**

One WSI feature and three immune marker genes were selected into the final recurrence-free survival (RFS) prediction model following the best integrated modeling framework. The resultant score showed a significantly improved prediction performance on the test dataset (mean time-dependent AUCs = 0.707) as compared to those of other types (e.g: mean time-dependent AUCs of AJCC tumor stage = 0.525) of input data integration. To assess that the risk score could provide a higher-resolution risk stratification, a lower-risk subgroup (or a higher-risk subgroup) was arbitrarily assigned according to score falling below (or above) the median score. The lower risk subgroup had significantly longer median RFS time than that of the higher-risk patients (median RFS = 903 vs. 265 days, log-rank test p-value< 0.0001). Additionally, the higher-risk subgroup, in contrast to the lower-risk patients were characterized with a significant downregulation of immune checkpoint genes, suppressive signal in tumor immune response pathways, and depletion of CD8 T cells. These observations for the higher-risk subgroup suggest that new targets for adoptive or checkpoint-based combined systemic therapies may be useful.

**Conclusion:**

We developed a novel prognostic model to predict RFS for HCC patients, using one feature that can be automatically extracted from routine histopathological images, as well as the expression profiles of three immune marker genes. The methodology used in this paper demonstrates the feasibility of developing prognostic models that provide both useful risk stratification along with valuable biological insights into the underlying characteristics of the subgroups identified.

## Author summary

Prognosis of recurrence events is important for HCC patients due to a high likelihood of post-surgery recurrence. Recurrence risk is related to both intra-tumor cell differentiation, and functional characteristics of the immune microenvironment. It remains under investigation as whether integrating quantitative whole slide image (WSI) features with the expression profile of immune marker genes can improve the risk stratification, and whether clinical outcome prediction can assist in understanding molecular biology that drives the outcome. By combining information from WSI features and the expression of immune marker genes, we derived a risk score, supported with underlying molecular mechanisms, that was shown to effectively stratify patients according to recurrence risk whereby the higher-risk subgroup experienced tumor recurrence events almost two years earlier than the lower-risk patients. The methods provide an effective way to build prognostic models with good performance and internal validity that are able to combine information from sets of features of different types.

## Introduction

Hepatocellular carcinoma is the third-leading cause of cancer-related deaths [1], and the high post-surgery recurrence rate remains a major obstacle for the efficacy of its curative treatments [2–5]. The RFS is defined as the shortest interval between the time of initial diagnosis and the time of first event occurrence. Previous studies attempted to use molecular subtyping to stratify prognostic risk. For example, in the first comprehensive study [6] regarding the TCGA data of HCC, the authors performed clustering analysis using the expressional profile of 66 representative immune marker genes on the cohort. They identified two clusters of tumor samples exhibiting ubiquitous high expression of these genes and four other clusters with relative low expression profile in overall. However, such distinct pattern in RNA expression profiles did not translate into a significant association with survival outcomes [6]. Moreover, the method utilized expression profile from a large panel of genes, which will be difficult for an affordable and reproducible clinical implementation. Taken together, these clusters were unable to be used as a predictor for risk stratification.

At least two possible reasons could explain such discrepancy. The first could be due to the unsupervised learning techniques used for cancer subtyping. Statistical learning techniques with variable selection and as supervised by clinical outcome of interest offer a simple yet usually more accurate model that have been successfully applied in previous studies [7–9]. Another reason was that the gene expression measured from bulk tissues might not be able to capture the complex functional characteristics of the tumor microenvironment. Single-cell profiling allows interrogation of transcriptomics from individual types of cells within tumor bulk but an unbiased delineation of cell types from high-dimensional sequencing data is still facing analytical challenges [10,11]. Addition of the histopathologic features from whole slides images (WSI) is one straightforward way to account for intra-tumor heterogeneity. One previous study showed that the immune cell compositions as characterized using multiplex immunohistochemistry could significantly explain prognostic variations of HCC patients [12]. Moreover, quantitative features describing the morphology or texture variations of tumor cells had also demonstrated their prognostic values in our and other studies [13–15].

In this study, we present an ensemble learning framework for predicting RFS of HCC patients using immune marker genes and WSI features. We used supervised survival modeling to specifically select immune marker genes that contributed most to the RFS of HCC. We also incorporated computer-aided processing of WSI features to adjust for tumor heterogeneity. We identified the best integrated modeling framework which generates multiple candidate models with a superior overall prediction performance. We then selected a representative risk score from this framework and evaluated its significance in RFS stratification. We finally explored its underlying molecular mechanisms related to a higher risk of recurrence development. Our methods provide an effective risk stratification for HCC recurrence and new immunotherapeutic insights.

## Methods

### Overall methodology

As illustrated by Fig A in S1 Appendix, the analyses and methods used in this study can be divided into five steps. Firstly, for input preparation, we curated the clinicopathological and clinical outcome variables to avoid missing and imbalanced data and processed the immune marker gene expression data from TCGA database. We also extracted and engineered imaging features from WSI to capture the nuclei and global tissue regions-related variations. In the second step, we applied supervised survival modeling to establish base as well as ensemble learners for RFS prediction. Then these trained models were tested by multiple test datasets resampled to assess the average prediction accuracy. Next, a simple model was selected from the best integrated modeling framework to compute the risk score. Its significance of recurrence risk stratification was evaluated on the whole study data. Finally, we explored the molecular mechanisms relevant to the higher risk of recurrence. This involves the identification of differentially expressed genes, enriched immune-related gene signal pathways and the deconvolution of immune cell compositions.

### Data source

The study used a retrospective cohort data of HCC patients from the Cancer Genome Atlas Liver Hepatocellular Carcinoma (TCGA-LIHC) project [6]. We included a total of 231 patients who had documentation of clinicopathologic information, follow-up data for tumor recurrence events, WSI data, and RNA sequencing data of the primary tumor tissue sample. The recurrence events include extrahepatic recurrence, intrahepatic recurrence, local recurrence and new primary tumor event, whichever comes first. Both death (n = 22) and lost follow-up (n = 86) were considered as the censoring events. We used the time of initial pathologic diagnosis as the starting time point.

The variables considered for analysis are as follows. We analyzed 8 clinicopathologic variables including age at initial diagnosis, body mass index (BMI), gender, race, history of HCC-related risk factors, surgical procedure, tumor grade and the American Joint Committee on Cancer (AJCC) pathologic tumor stage. The cirrhosis variable was not included for model development due to a large number of missing values (54.1%). The distribution of these variables was summarized in Table A in S1 Appendix. A curated list of 66 cell surface marker genes representing different immune cell populations used in a previous study [6] was shown in Table B in S1 Appendix. The selection of the 66 immune marker genes was based on a review of published functional studies on cohorts independent from the one used in this study. Furthermore, the selection of these immune markers was conducted independently and prior to analyses performed on the study cohort. For RNA sequencing data, we used the fragments per kilobase of exon model per million reads mapped (FPKM) measurements to represent gene expression level. The FPKM values plus 1 were log transformed before being used for model fitting.

### WSI processing

H&E stained WSI diagnostic slides with 40X magnification were used for image feature extraction. We used the OTSU method [16] to segment the stained tissue region and cropped a block of 32,000 x 32,000 pixels as the foreground region. We shrunk this block to a size of 1,000 x 1,000 pixels to represent the global region of interest (ROI) for feature extraction. We then extracted a total of 18 gray level-related features from the region.

For nuclei-related feature extraction, we first randomly sampled 20 blocks with a size of 1,000 x 1,000 pixels from the foreground region, and then performed nuclei segmentation with following major steps: 1) using color deconvolution [17] to convert the color space from RGB to HEO space; 2) using the OTSU method [16] to segment nuclei regions using Laplacian filtered H channel; 3) optimizing the shape of segmented regions by adaptive threshold segmentation [18], morphological operation, and by fitting an ellipse and convex hull. For each block, we randomly sampled 20 nuclei as the nuclei ROI for feature extraction. We computed a total of 135 features related to the shape of nucleus. In particular, for each feature, we calculated the ratio of its minimum to the maximum (min-max rate, mrate) among all nuclei sampled from one WSI to represent contrast or variations of such shape feature. Image analysis was performed with Python 3.7.6 and packages including “Opencv” [19] and “Pyradiomics” [20].

### Survival model development

The least absolute shrinkage and selection operator (Lasso)-based Cox models [21] was used to select predictive features from high-dimensional input data under supervised framework for RFS outcome. Risk scores was computed from the linear combination of features and their coefficients. Twenty random splits of the cohort into training dataset (66.7%, 154 patients) and test dataset (33.3%, 77 patients) were performed. For each split the C-index [22] was used as the metric to assess prediction accuracy. A value of 1 in C-index measurement indicates perfect prediction while 0.5 for random guess. Across the 20 splits a final model was selected as the simplest model from among those with a C-index between the median and top 25^th^ quartile.

To assess the prediction significance, the model development described above was repeated for 100 times, with each time based on the data where the correspondence of input covariates to RFS outcome was randomly reshuffled. A normal distribution was fitted to the null distribution and the p-value was computed for the proposed model. Prediction accuracy at specific time points or time intervals were estimated by computing time-dependent AUCs [23] for candidate models. The time-dependent receiver operating characteristics (ROC) measure the sensitivity and specificity of a continuous risk score for predicting a clinical outcome that can be time-dependent, with a value of AUC of 1 indicates a perfect prediction while 0.5 represents a random guess. The model development and comparison were performed by R version 4.1.3.

Following standard machine learning model selection procedure, the 3-fold cross validation performed within training datasets was used to tune Lasso penalty parameter. To mitigate the lack of randomness due to single split for training and test dataset, we performed 20 random splits which result in 20 trained models under same type of input data integration framework. These 20 models have different Lasso penalties and hence different sets of selected features/variables. The mean value of 20 evaluation metrics estimates derived based on test dataset was computed to rank data integration in terms of RFS prediction performance.

The final risk score model was selected arbitrarily as the simplest model among those with a C-index between the median and top 25^th^ quartile. This is to make sure that our selected model approximates the average performance of the identified modeling technique, while at the same time constrained the model complexity.

As a foundation of comparison for model performance, single domain features (Table D in S1 Appendix) such as AJCC tumor stage, clinicopathologic variables, image features, or immune marker genes expression as input variables were firstly tested. More advanced data integration based on these single domains were then attempted. The integration can be either via a pairwise combination of individual type of features, or via a new model using the risk scores already developed from single domain features (Table D in S1 Appendix).

### Analysis of immune phenotyping variations

For differential gene expression analysis, the negative binomial generalized linear model with tag-wise dispersion in R package edgeR [24] was applied. The raw count data was normalized by the TMM (the trimmed mean of M values) [25]. Genes whose mean of counts was more than 15 reads and with non-zero count in every sample were retained for normalization which resulted in a total of 13,709 genes used in downstream analysis. Pathway enrichment analyses was performed using multiple algorithms including GOseq and GSEA [26–28].

The CIBERSORTx software was used to deconvolve the relative fractions of different immune cell types from the RNA sequencing data [29,30]. To infer the significance of enrichment of cell types between the higher and lower-risk patient subgroups, we used Wilcoxon rank-sum test to compute p-values. The Benjamini-Hochberg procedures were applied to obtain the adjusted p-values [31] and to control the false discovery rate (FDR).

## Results

### Descriptive summary of study datasets

A total of 123 patients (53.2%) had a tumor recurrence since initial diagnosis (Fig B in S1 Appendix), with a median RFS of 498 days (95% CI: 398, 658 days). The distribution of clinicopathologic variables was summarized in Table A in S1 Appendix. Only race had an association with RFS that was borderline significant (adjusted p-value = 0.018) after correcting for multiple testing, suggesting the lack of prognostic prediction power of these conventionally used variables. For immune marker genes, 6 genes were significantly associated with RFS under univariate survival models (Table C in S1 Appendix). The genes *IL12A*, *IL13* were positively associated with recurrence risk, while the remaining 4 genes had a negative correlation.

### Development of integrated RFS prediction model

**Fig 1:**
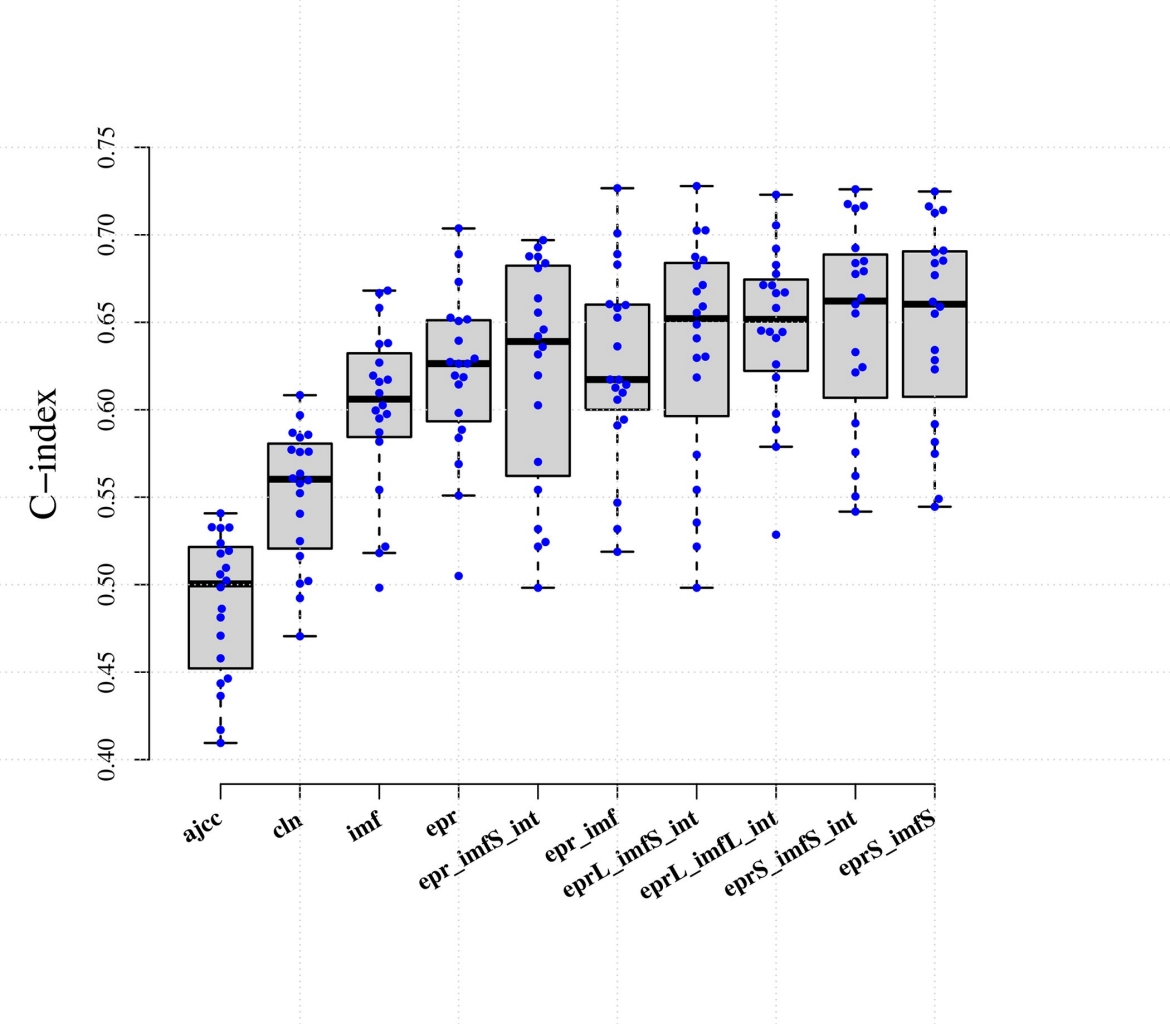
Summary of C-index on test datasets generated from 20 repetitions of 10 different input data integrations. Abbreviations can be referred to Table D in S1 Appendix.

To develop the best prediction model using the three different types of input data, we performed data integrations and assessed the prediction accuracy using the same evaluation metrics and survival modeling framework (Fig 1 & Table D & E in S1 Appendix). We noted that the AJCC tumor stage commonly used for the prognosis of overall survival had low prediction efficacy (mean C-index = 0.488). In contrast, many image features used as single predictors were more predictive of RFS as compared to individual clinicopathologic variables (mean C-index = 0.601 vs. 0.552). Models solely using immune marker genes expression as input had slightly higher accuracy when compared to those based on image features alone (mean C-index = 0.621 vs. 0.601), suggesting that combining both types of features may be useful for predicting RFS.

We next investigated the prognostic benefits of adding image features to the expression profile of immune markers. The improvement of mean C-index was limited (mean C-index = 0.626 vs. 0.601) if combining all variables for model training. However, when integrating the risk scores respectively developed from image and immune markers, we observed an obviously higher average C-index on the test dataset (mean C-index = 0.650 vs. 0.601). Introducing either interaction variables or clinicopathologic variables showed no improvement on the prognostic accuracy (Fig C in S1 Appendix & Table E in S1 Appendix). Fitting by more flexible model class like random survival forest model, which accounts for possible non-linearity in predictors, did not show increase in accuracy (mean C-index = 0.604, Fig D in S1 Appendix). We thus considered those combining image-based risk score with immune markers-based score as the best type of data integration.

We further showed via permutation analysis that this gain of C-index was not due to chance (p-value<0.001), as no model demonstrated predictive capacity (mean C-index = 0.503) on the data where the underlying pattern had been removed by randomly re-ordering the covariates and RFS outcome (Fig E in S1 Appendix).

### Characteristics of the risk score

A final risk score model with a C-index (0.677) close to the average performance (Fig 1) was selected as representative of a good model integrating information from both image and gene expression. This final risk score was comprised of one risk score based on one image feature and one score based on expression of three genes (Table F in S1 Appendix). The image feature included in the score specifically measured the contrast in the shape and size of nuclei sampled from WSI, with a smaller value representing a higher level of variation of the nuclei morphology. The feature value was negatively associated with recurrence risk. For the immune markers, two genes (*HLA-DRB1* and *CD4*) were negatively associated with recurrence hazard, while one gene (*IL12A*) had a positive correlation.

The distribution of the risk score for all subjects is shown in Fig F in S1 Appendix. Using this score as an independent variable yields a Cox model with a computed p-value <0.001, suggesting a strong fit and positive correlation of the score and RFS. Under the derived Cox model, the risk of developing recurrence increases 1.913 times with a one unit increase of the score.

**Fig 2:**
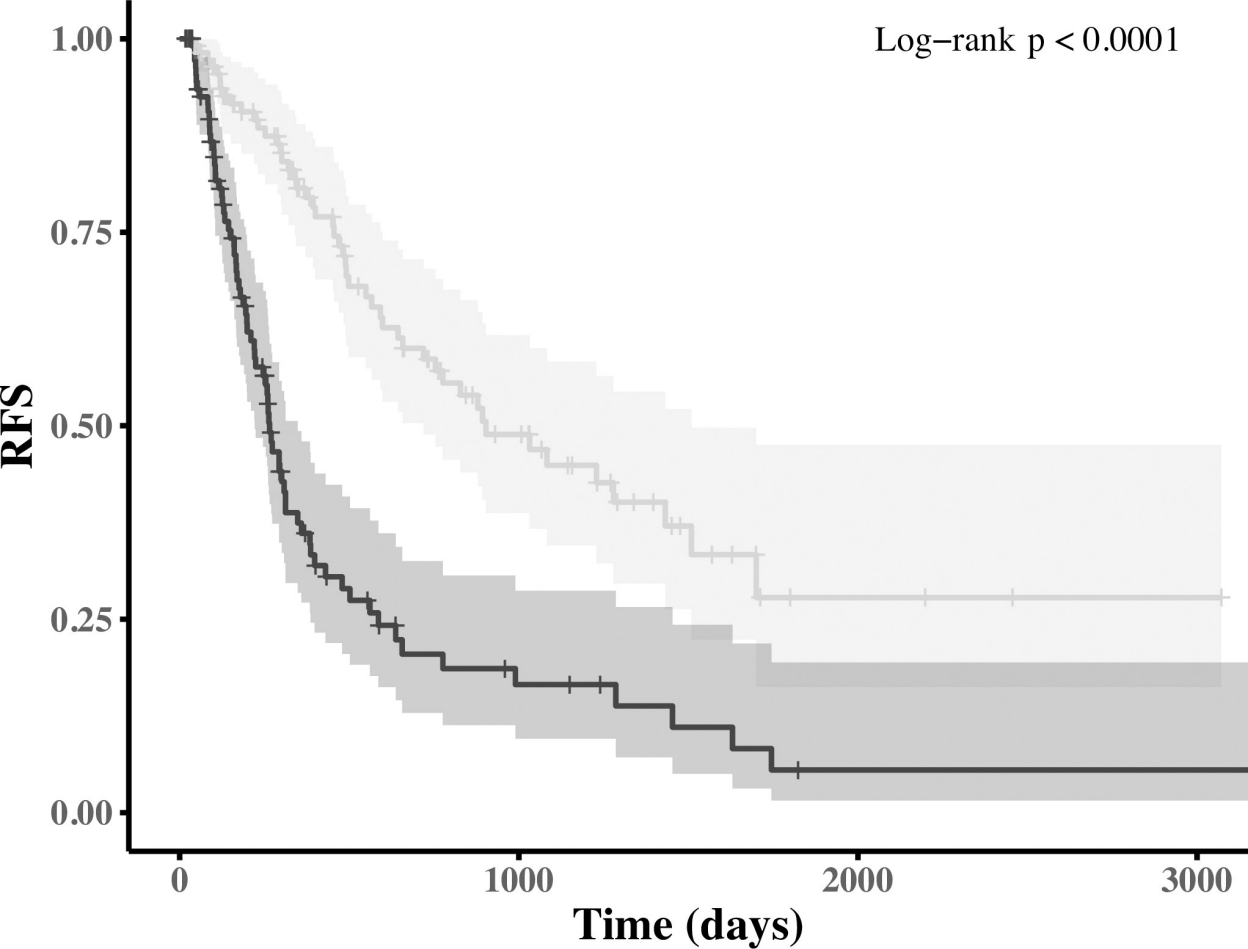
Kaplan-Meier analysis of RFS for the higher and lower-risk subgroups as stratified by the proposed risk score. The shaded area indicates the 95% confidence interval.

Subjects were stratified into lower (at or below the median score) and higher-risk subgroups (above the median score). As shown in Fig 2, the score could effectively identify higher-risk patients (median RFS = 265 days, 95% CI: 221, 348 days) from the lower-risk ones (median RFS = 903 days, 95% CI: 719~1509 days), with a difference of around 21 months in median RFS (log-rank test p-value<0.0001). Moreover, the selected risk score has a significantly higher time-varying prediction accuracy when compared to the AJCC tumor stage on the test dataset (Fig G in S1 Appendix, mean time-dependent AUCs = 0.707 vs. 0.525, p-value<0.0001).

### Differential risk-related immune phenotyping variations

The differential gene expression analyses between the higher-risk (n = 115) and lower-risk (n = 116) subgroups were conducted to explore potential differences in gene signatures. There were 1,418 significantly upregulated genes and 937 downregulated genes among the higher-risk compared to the lower-risk subgroup patients (Fig H in S1 Appendix). Notably, among the 66 immune marker genes (Table B in S1 Appendix), 19 were differentially expressed among the two subgroups, of which only one gene *ARG2*, a marker gene of myeloid derived suppressor cells (MDSCs), was over-expressed in the higher-risk subgroup (Table G in S1 Appendix). The immune marker genes significantly downregulated in higher-risk patients include T cell marker genes *CD8A*, *CD3E*, *CD247* (*CD3Z* or *TCRZ*), *CD3G*, *CD3D* and *CD4*, immune checkpoint stimulatory genes *CD27*, *CD86* and *CD28* and immune checkpoint inhibitory genes *PDCD1LG2* (*PD-L2*), *LAG3*, *CD274* (*PD-L1*), *HAVCR2 (TIM3)*.

To further explore potential underlying molecular mechanisms for the observed differences in gene expression, we performed the GO and gene sets enrichment analysis (GSEA). Among higher-risk patients, the downregulated genes were significantly enriched in adaptive immune response GO terms or pathways (Fig IA & J in S1 Appendix), while the upregulated genes were over-represented in chromosome segregation, cell cycle regulation and DNA repair GO terms or pathways (Fig IB & K in S1 Appendix).

Computational deconvolution of RNA sequencing data (Fig L in S1 Appendix) was applied to investigate the composition of infiltrating immune cells. The inferred relative fractions of immune cell varied within and potentially across risk subgroups (Fig LA in S1 Appendix). Between the higher-risk and lower-risk subgroup, there was a significant depletion of CD8 T cells and enrichment of tumor associated macrophages (TAMs) (Fig LB in S1 Appendix). This observation is indicative of transition from a more permissive to suppressive status of HCC tumor immune microenvironment for the higher-risk subgroup compared to the lower-risk subgroups.

## Discussion

In this study, a novel prognostic model for RFS prediction was developed by integrating scores independently trained from 66 immune marker genes and 153 texture features automatically extracted from WSI. The model achieved improved prediction accuracy as compared to ones based on image features or clinicopathologic variables alone. A representative risk score selected from the proposed modeling consisted of only four input variables, with one from image feature and another three from immune markers. The score was a strong independent predictor for RFS and could effectively identify patients with a relatively higher risk of developing recurrence events (log-rank test p-value< 0.001). We discovered that those higher-risk subgroup patients showed a significant enrichment of immune resting and suppressive signals. Our model, with only four features and with their contribution to RFS prediction delineated, can help facilitate potential clinical interpretation and translation.

Our method has improved the reliability in potential clinical application of multi-modality data-based risk prediction model. Prognosis of recurrence events is particularly important for HCC patients due to a high likelihood of post-surgery recurrence [4,5,32–34]. Traditional methods like AJCC tumor staging may not adequately account for of recurrence risk due to the high level of heterogeneity inherent in HCC [35–37]. Recurrence risk is driven by both intra-tumor cell morphology, and functional characteristics of the immune microenvironment [5,6,12,38,39]. By combining information from WSI features and the expression of immune marker genes, we derived a risk score that was shown to effectively stratify patients according to recurrence risk whereby the higher-risk subgroup experienced tumor recurrence events almost two years earlier than the lower-risk patients. To our knowledge, this is the first study to specifically investigate the combinatorial effect of computer-aided WSI processing and immune marker gene expression for HCC’s RFS prediction.

From molecular biology side, exploratory comparisons between a higher recurrence risk subgroup and lower recurrence risk subgroup indicated differences in immune related phenotypes of tumors at both cellular and molecular levels. Digital cytometry of immune cell composition showed a significant deprivation of CD8 T cells and infiltration of TAMs among higher-risk patients. This is consistent with previous studies which demonstrated that the tumor progression is closely related to the lack of effector lymphocytes and to the accumulation of immunosuppressive cell types like TAMs [5,40]. Molecularly, the immune marker gene *ARG2* was identified to be over expressed in the higher-risk subgroup. The *ARG2* has been reported previously to be correlated with compromised T lymphocyte proliferation and function due to increased level of arginase [41–44]. Another recent study using clinical samples showed that *ARG2* was significantly upregulated in primary and recurrent tumor samples compared to normal tissues and associated with HCC progression [45]. In our cohort analyses, both stimulatory and inhibitory immune checkpoint genes were down-regulated in the higher-risk subgroup, pointing to a more intricate hypothesis for consideration regarding molecular factors underlying the interplay of immune regulation that may affect immunotherapy response and the risk of recurrence. These observations were complemented by enrichment analysis, where the genes over-expressed in the higher-risk subgroup were significantly represented in pathways related to chromosomal aberrations, cell proliferation and DNA damage response. Collectively, our results confirmed that an immune ‘cold’ phenotype was highly related to a poorer RFS, while an active tumor immunity was associated with a superior RFS.

Our study produced an HCC recurrence risk stratification with some between-subgroup characteristics that are reminiscent to those reported by other subgrouping analyses in the literature. For example, a recently developed HCC immune classification system introduced an ‘Inflamed’ and a ‘Non-inflamed’ class [5,46]. The ‘Inflamed’ class was considered as potential responders to immune-checkpoint inhibitors (ICIs). For the ‘Non- inflamed’ class, genetic analysis revealed that there are increasing levels of chromosomal instability, deletion in genes related to antigen-presenting machinery, and a higher frequency of oncogenic driver mutations, all of which would lead to primary resistance to immunotherapies. Based on the features of immune cells infiltration and related molecules expression pattern, our higher-risk subgroup was similar to the ‘Non-inflamed’ class, while the lower-risk subgroup approximated the ‘Inflamed’ class.

The clinical trials in advanced-stage HCC patients have already demonstrated a synergistic advantage in overall survival when combining ICIs with anti-angiogenic antibodies over standard of treatment regimens [47–49]. It was reported that several outcomes could emerge following the administration of anti-angiogenic antibodies, which include tumor vascular normalization that leads to improved drug delivery, remodeling of the immunosuppressive environment through regulating TAMs, MDSCs and Tregs, and blockade of tumor-intrinsic pathways, such as MAPK, WNT–β-catenin, CDK4/6 and/or PI3K–PTEN signaling, that cause immune cell exclusion [5]. Under this adjusted context on tumor microenvironment, the ICIs treatment taken concurrently could successfully facilitate activation and infiltration of various anti-tumor immune cells such as CD8 T cells, DCs, and NK cells, and transform TAMs to an anti-tumor M1 macrophage phenotype. We note that the ICI atezolizumab and the anti-angiogenic agent bevacizumab combinations have already been recommended as the first line therapy for the patients with advanced-stage HCC [5]. It would be of interest to evaluate the RFS from higher-risk patients who have received such combination therapies. In addition, adoptive immunotherapies like engineering chimeric antigen receptor T cells (CAR-T) might be one option of treating the higher-risk subgroup patients via restoring their tumor immune microenvironment. A recent Phase I trial reported anti-tumor activity of CAR-GPC3 T cells in patients with advanced HCC with well-controlled adverse effect [50]. Further progress in HCC clinical trials may benefit from studies of novel approaches to the treatment of higher-risk subgroup that are informed by the identification of specific subgroup characteristics associated with response or risk using methods as demonstrated by this study.

Our observational study has several limitations. First, we did not include adjuvant therapy data into model development because for a large number of patients (>20%) these data are missing. Incorporating treatment information can improve our model by enabling control for treatment-based confounding especially given that use of investigational drug could possibly contribute to RFS [5,51]. Also, having a more complete set of other clinicopathologic variables, such as the size and number of tumors, or vascular invasion status, could potentially improve our model. Second, the correspondence between the sample used for diagnosis and that was used for sequencing was unknown, however the time interval between diagnosis and tissue sample procurement was small [52]. Third, the TCGA data used was generated on various Illumina platforms and from different local labs. Fourth, while a randomized assignment of subjects to training makes for most representative subset of the available data for training purposes, it has the disadvantage of creating a test set that is very similar to the training set including potential confounders. Consequently, external validation is needed prior to clinical translation of these findings. Lastly, for the comparison of RFS prediction model, we did not perform statistical testing on the difference in C-index due to the concern that C-indices we computed from the test datasets resultant from 20 splits of cohort data is more like repeated measures because the test datasets can possibly overlap with each other. Statistical testing adjusted for the independence violation needs to be further explored but is out of scope of this study.

Notwithstanding with these limitations, we believe the methods described provide an effective way to build prognostic models with good performance and internal validity that are able to combine information from sets of features of different types.

## Supporting information

S1 Appendix Supporting tables and figures. (DOCX)

S1 File The covariance matrix of 66 immune marker genes. (CSV)
