# Supplementary material for: Recurrence risk stratification of hepatocellular carcinomas based on immune gene expression and features extracted from pathological images
Source: PLoS Comput Biol. 2023 Dec 29;19(12):e1011716. doi: 10.1371/journal.pcbi.1011716 (PMC10783785; doi:10.1371/journal.pcbi.1011716)
Supplement: S1 Appendix — (DOCX) [file pcbi.1011716.s001.docx]

**Supplementary materials**

**Recurrence risk stratification of hepatocellular carcinomas based on immune gene expression and features extracted from pathological images**

Contents

[Supplementary Tables 1](#_Toc42678028)

[Supplementary Figures 7](#_Toc42678029)

[Supplementary Methods 10](#_Toc42678030)

[Reference 12](#_Toc42678031)

# Supplementary Tables

Table A: Summary of distributions of clinicopathologic variables and their association with recurrence-free survival of the study cohort.

| Characteristics | Summary | RFS association p-value † |
| --- | --- | --- |
| Age at diagnosis | 231 (100%) | 0.367 |
| *mean/sd* | 57.5/12.7 | / |
| BMI | 231 (100%) | 0.064 |
| *mean/sd* | 25.9/9.2 | / |
| Cirrhosis | 106 (45.9%) | 0.415 |
| *Yes* | 76 (32.9%) | / |
| *No* | 30 (13.0%) | / |
| Gender (male) | 231 (100%) | 0.286 |
| *male* | 168 (72.7%) | / |
| *female* | 63 (27.3%) | / |
| Race | 231 (100%) | 0.018 |
| *Asian* | 122 (52.8%) | **/** |
| *White* | 103 (44.6%) | **/** |
| History of HCC risk factors | 231 (100%) | 0.286 |
| *With alcohol consumption* | 76 (32.9%) | / |
| *With hepatitis B* | 84 (36.3%) | / |
| *With hepatitis C* | 31 (13.4%) | / |
| *With other risk factor* | 24 (10.4%) | / |
| *No risk factor* | 55 (23.8%) | / |
| Surgical procedure | 231 (100%) | 0.420 |
| *Lobectomy* | 89 (38.5%) | / |
| *Extend lobectomy* | 13 (5.6%) | / |
| *Segmentectomy multiple* | 53 (22.9%) | / |
| *Segmentectomy single* | 64 (27.7%) | / |
| Tumor grade | 231 (100%) | 0.517 |
| *G1* | 32 (13.9%) | / |
| *G2* | 107 (46.3%) | / |
| *G3 or G4* | 92 (39.8%) | / |
| AJCC pathologic tumor stage | 231 (100%) | 0.517 |
| *Stage I* | 119 (51.5%) | / |
| *Stage II* | 54 (23.4%) | / |
| *Stage III & IV* | 58 (25.1%) | / |

† The p-values were adjusted for multiple testing using FDR procedure.

Table B: The 66 immune marker genes used in the study.

| **Gene symbol** | **Representing cells or functions** |
| --- | --- |
| CD19 | B cells |
| MS4A1 | B cells |
| FOXP3 | Tregs |
| IL2RA | Tregs |
| LRRC32 | Tregs |
| TGFB1 | Tregs |
| HLA-A | MHC I |
| HLA-B | MHC I |
| HLA-C | MHC I |
| HLA-DMA | MHC II |
| HLA-DMB | MHC II |
| HLA-DOA | MHC II |
| HLA-DOB | MHC II |
| HLA-DRB1 | MHC II |
| ARG1 | Myeloid derived suppressor cells |
| ARG2 | Myeloid derived suppressor cells |
| CD33 | Myeloid derived suppressor cells |
| CSF2 | Myeloid derived suppressor cells |
| CSF3 | Myeloid derived suppressor cells |
| IDO1 | Myeloid derived suppressor cells |
| IL10 | Myeloid derived suppressor cells |
| IL13 | Myeloid derived suppressor cells |
| IL1B | Myeloid derived suppressor cells |
| IL4 | Myeloid derived suppressor cells |
| IL6 | Myeloid derived suppressor cells |
| ITGAM | Myeloid derived suppressor cells |
| CD274 | CheckPoint |
| CD276 | CheckPoint |
| CD28 | CheckPoint |
| CD80 | CheckPoint |
| CD86 | CheckPoint |
| CTLA4 | CheckPoint |
| HAVCR2 | CheckPoint |
| LAG3 | CheckPoint |
| LGALS9 | CheckPoint |
| PDCD1 | CheckPoint |
| PDCD1LG2 | CheckPoint |
| VTCN1 | CheckPoint |
| CD247 | T cell coreceptor |
| CD3D | T cell coreceptor |
| CD3E | T cell coreceptor |
| CD3G | T cell coreceptor |
| CD4 | T cell coreceptor |
| CD8A | T cell coreceptor |
| CD8B | T cell coreceptor |
| CD27 | Activator |
| CD70 | Activator |
| ICOS | Activator |
| ICOSLG | Activator |
| TNFRSF4 | Activator |
| TNFRSF9 | Activator |
| TNFSF9 | Activator |
| GZMA | Effector protein |
| GZMB | Effector protein |
| PRF1 | Effector protein |
| IFNG | Activating cytokine |
| IL12A | Activating cytokine |
| IL12B | Activating cytokine |
| IL2 | Activating cytokine |
| LTA | Activating cytokine |
| TNF | Activating cytokine |
| KIR2DL1 | NK receptor |
| KIR2DL4 | NK receptor |
| KIR2DS4 | NK receptor |
| KIR3DL1 | NK receptor |
| KIR3DL2 | NK receptor |

Table C: Summary of immune marker genes significantly associated with RFS.

| Gene symbols | Coefficients under univariate Cox model | Adjusted p-value |
| --- | --- | --- |
| CD4 | -0.349 | 0.011 |
| HLA-DRB1 | -0.352 | 0.011 |
| CD3E | -0.338 | 0.011 |
| PRF1 | -0.380 | 0.011 |
| IL12A | 0.253 | 0.024 |
| IL13 | 0.200 | 0.039 |

Table D: Summary of input data integrations.

| Model abbreviations | Included features |
| --- | --- |
| ajcc  cln  epr | The AJCC tumor stage variable;  Lasso selected clinicopathologic variables;  The expression profile of immune marker genes (n=66); |
| imf | WSI features (n=153); |
| epr_imf | 66 immune marker genes and 153 WSI features; |
| eprS_imfS | One immune marker genes-based risk score and one WSI features-based risk score; |
| eprS_imfS_int | One immune marker genes-based risk score and one WSI features-based risk score, and their interaction; |
| eprL_imfL_int | Lasso selected immune marker genes, lasso selected WSI features, and their interaction; |
| epr_imfS_int | 66 immune marker genes, one WSI features-based risk score, and their interaction; |
| eprL_imfS_int | Lasso selected immune marker genes, one WSI features-based risk score, and their interaction; |
| imf_cln | 153 WSI features and 8 clinicopathologic variables; |
| epr_cln | 66 immune marker genes and 8 clinicopathologic variables; |
| epr_imf_cln | 66 immune marker genes, 153 WSI features and 8 clinicopathologic variables; |
| eprS_imfS_cln | One immune marker genes-based risk score, one WSI features-based risk score and 8 clinicopathologic variables; |
| epr_imf_cln_RSF | 66 immune marker genes, 153 WSI features and 8 clinicopathologic variables, using random survival forest model. |

Table E: Summary of C-index from 20 repetitions of models with different input data integrations.

| **Model** | **mean** | **std** |
| --- | --- | --- |
| ajcc | 0.488 | 0.041 |
| cln | 0.552 | 0.038 |
| imf | 0.601 | 0.048 |
| epr | 0.621 | 0.047 |
| epr_imfS_int | 0.621 | 0.065 |
| epr_imf | 0.626 | 0.054 |
| eprL_imfS_int | 0.635 | 0.065 |
| eprL_imfL_int | 0.646 | 0.047 |
| eprS_imfS_int | 0.649 | 0.059 |
| eprS_imfS | **0.650** | 0.058 |
| imf_cln | 0.587 | 0.056 |
| epr_cln | 0.621 | 0.029 |
| epr_imf_cln | 0.634 | 0.040 |
| eprS_imfS_cln | 0.641 | 0.050 |
| epr_imf_cln_RSF | 0.604 | 0.049 |

Table F: Summary of the selected risk score. The feature weight was applied to the features already scaled with mean and standard deviation.

| **Input feature type** | **Features** | **Feature weight** |
| --- | --- | --- |
| Image | nu_shape_cvx_smoothness_mrate | -0.335 |
| Immune marker | HLA-DRB1 | -0.428 |
|  | CD4 | -0.045 |
|  | IL12A | 0.411 |

#

Table G: Summary of differentially expressed immune marker genes†. A positive logFC value represents the gene was over-expressed in the higher-risk subgroup.

|  | **EntrezID** | **logFC** | **logCPM** | **FDR adjusted p-value** |
| --- | --- | --- | --- | --- |
| HLA-DRB1 | 3123 | -1.4679955 | 8.04188248 | 8.95E-14 |
| GZMA | 3001 | -1.3284035 | 2.4660834 | 3.98E-10 |
| CD247 | 919 | -0.9852453 | 1.95073304 | 6.41E-09 |
| PRF1 | 5551 | -1.0871797 | 2.94381185 | 2.40E-08 |
| CD3E | 916 | -1.1342427 | 3.28211697 | 7.50E-08 |
| GZMB | 3002 | -1.186006 | 1.24849608 | 3.96E-07 |
| CD4 | 920 | -0.7703563 | 5.63230656 | 1.00E-06 |
| HLA-DMB | 3109 | -0.9431364 | 4.55179399 | 7.52E-07 |
| PDCD1LG2 | 80380 | -0.9784969 | 0.49343052 | 2.09E-06 |
| CD86 | 942 | -0.7887041 | 2.36722756 | 2.64E-06 |
| HLA-B | 3106 | -0.8803111 | 10.8224883 | 2.15E-06 |
| CD8A | 925 | -1.1865822 | 2.91219727 | 6.06E-06 |
| HLA-DMA | 3108 | -0.9602213 | 6.23514854 | 7.03E-06 |
| CD27 | 939 | -1.1292299 | 1.35323309 | 1.29E-05 |
| HLA-DOA | 3111 | -0.9791077 | 3.65833007 | 1.02E-05 |
| CD3G | 917 | -0.9430447 | 0.82966967 | 2.91E-05 |
| CD274 | 29126 | -0.7275445 | 1.12163821 | 0.00013359 |
| CD33 | 945 | -0.6458145 | 1.22278979 | 0.00014549 |
| HAVCR2 | 84868 | -0.6561281 | 2.76225754 | 0.00022554 |
| HLA-C | 3107 | -0.5759722 | 10.3640499 | 0.00026301 |
| CD3D | 915 | -0.7858755 | 2.43600148 | 0.00216922 |
| LAG3 | 3902 | -0.7987121 | 2.54254808 | 0.00285023 |
| HLA-A | 3105 | -0.4327335 | 10.4856808 | 0.01152832 |
| CD28 | 940 | -0.3956972 | 1.15074645 | 0.02830134 |
| LRRC32 | 2615 | -0.4153673 | 4.88817336 | 0.03678397 |
| ARG2 | 384 | 0.71095016 | 2.66622156 | 0.03423338 |
| FOXP3 | 50943 | -0.6067422 | 2.5501810 | 0.04857154 |

† The marker gene IL12A was filtered out prior to DE analysis due to relatively low expression.

Table H: Summary of extracted image feature names.

| Class | Feature names | Summary methods |
| --- | --- | --- |
| Global tissue slide features | 10Percentile, 90Percentile, Energy, Entropy, InterquartileRange, Kurtosis, Maximum, MeanAbsoluteDeviation, Mean, Median, Minimum, Range, RobustMeanAbsoluteDeviation, RootMeanSquared, Skewness, TotalEnergy, Uniformity, Variance | / |
| Nuclei shape features | mean_radius, perimeter, area, equivalent_diameter, compactness, roundness, auxiliary_circularity, amendment_circularity, regularity, extent_ratio, solidity, ellipse_short_axis, ellipse_long_axis, ellipse_angle, ellipse_foci, ellipse_eccentricity, ellipse_ellipticity, unnorm_smoothness, norm_smooth | mean, standard deviation, range, disorder, min-max rate |

# Supplementary Figures

Fig A: Summary of study workflow.


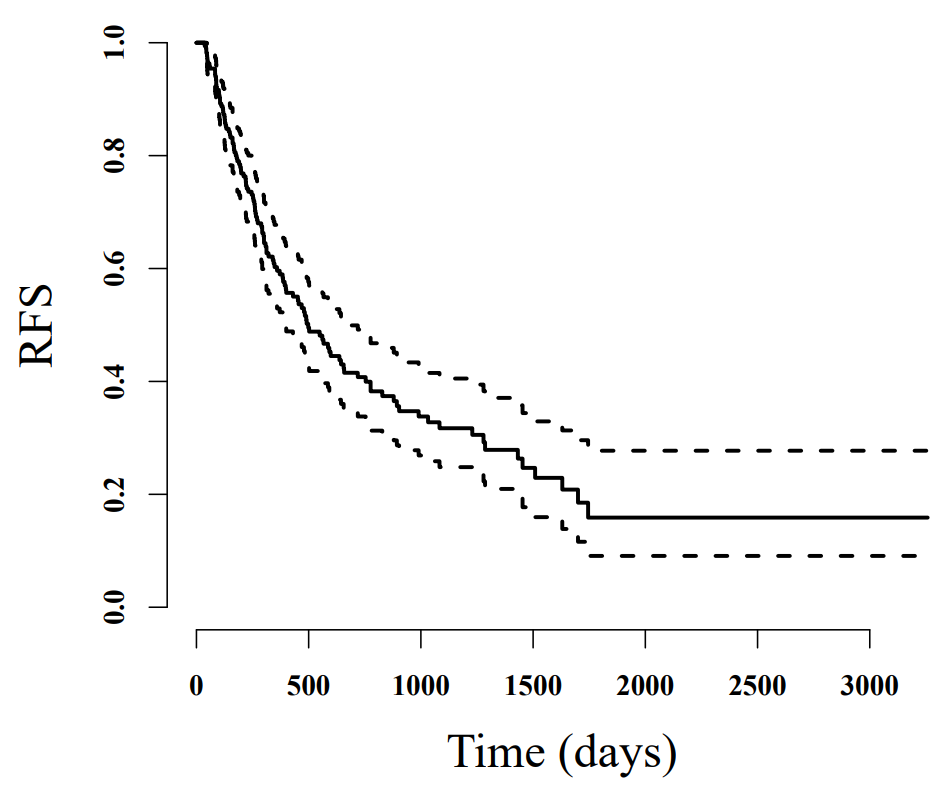


Fig B: RFS distribution of the study cohort. The dash line is the 95% confidence interval.

Fig C: Summary of C-index on test datasets generated from 20 repetitions of clinicopathologic variables-based input data integrations. Abbreviations can be referred to Table S4.

Fig D: Summary of C-index on test datasets generated from 20 repetitions of clinicopathologic variables, immune marker genes, and imaging features-based input data integrations, using random survival forest modeling. Abbreviations can be referred to Table S4.

Fig E: Summary of C-index on test datasets generated from 100 trials of 20 repetitions of 10 different input data integrations on randomly reshuffled dataset. The blue horizontal line represents the median C-index (0.650) generated from proposed modeling framework.


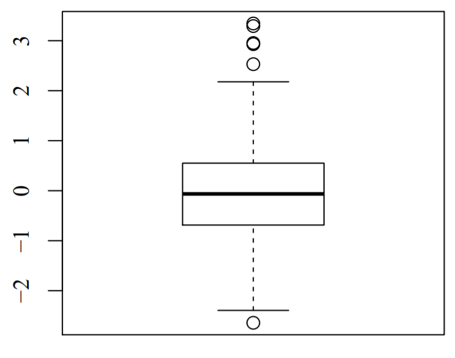


Fig F: Distribution of the selected risk score computed from the study cohort.

Fig G: Comparison of time-dependent AUCs of proposed risk score and AJCC stage.

Fig H: Analysis results of differential gene expression between the higher and lower-risk subgroup.

A

B

Fig I: Top 20 enriched GO biological pathways for genes downregulated (A) or upregulated (B) in higher-risk patients as relative to that of lower-risk subgroup.

A B

C D

E F

Fig J: Gene set enrichment analysis (GSEA) identified that immune response-related pathways were significantly enriched in lower-risk subgroup patients.

A B

Fig K: Gene set enrichment analysis (GSEA) identified that cell cycle-related pathways were significantly enriched among higher-risk subgroup patients.

A

B

Fig L: Barplot summary of inferred relative fractions of cell types (A) and volcano plot summary for the significance of difference in immune cellular compositions between the higher and lower-risk subgroup patients (B). A negative Z score in the volcano plot indicates the depletion of cell types in the higher-risk subgroup.

# Supplementary Methods

**Model development – cross validation and 20 data splits**

Following standard machine learning model selection procedure, the 3-fold cross validation performed within training datasets was used to tune Lasso penalty parameter. To mitigate the concern of chance of single data split into overly good or bad training and test dataset, we performed 20 splits and generated 20 trained models with same type of input data integration. These 20 models have different Lasso penalties and hence different sets of selected features/variables. The mean value of 20 evaluation metrics estimates derived based on test dataset was computed to determine which type of data integration provides more effective RFS prediction. The final risk score model was selected arbitrarily as the simplest model among those with a C-index between the median and top 25^th^ quartile. This is to make sure that our selected model approximates the average performance of the identified modeling technique, while at the same time constrained the model complexity.

**Image processing and feature extraction**

We used the OTSU method to segment the stained tissue region and cropped a block of 32,000 x 32,000 pixels as the foreground region. We shrunk this block to a size of 1,000 x 1,000 pixels to represent the global region of interest (ROI) for feature extraction. We then extracted a total of 18 gray level-related features from the region. For nuclei-related feature extraction, we first randomly sampled 20 blocks with a size of 1,000 x 1,000 pixels from the foreground region, and then performed nuclei segmentation with following major steps: 1) using color deconvolution[1] to convert the color space from RGB to HEO space; 2) using the OTSU method[2] to segment nuclei regions using Laplacian filtered H channel; 3) optimizing the shape of segmented regions by adaptive threshold segmentation[3], morphological operation, and by fitting an ellipse and convex hull. For each block, we randomly sampled 20 nuclei as the nuclei ROI for feature extraction. We computed a total of 135 features related to the shape of nucleus. In particular, for each feature, we calculated the ratio of its minimum to the maximum (min-max rate, mrate) among all nuclei sampled from one WSI to represent contrast or variations of such shape feature. Image analysis was performed with Python 3.7.6 and packages including “Opencv”[4] and “Pyradiomics”[5]. The feature names are listed in Table H in S1 Appendix.

# Reference

1. Ruifrok AC, Johnston DA: **Quantification of histochemical staining by color deconvolution.** *Anal Quant Cytol Histol* 2001, **23:**291-299.
2. Otsu N. A threshold selection method from gray-level histograms. IEEE transactions on systems, man, and cybernetics. 1979;9(1):pp.62-6.
3. Abdolhoseini M, Kluge MG, Walker FR, Johnson SJ. Segmentation of Heavily Clustered Nuclei from Histopathological Images. Sci Rep. 2019;9(1):4551.
4. Druzhkov PN, Erukhimov, V.L., Zolotykh, N.Y., Kozinov, E.A., Kustikova, V.D., Meerov, I.B. and Polovinkin, A.N.: **New object detection features in the OpenCV library.** *Pattern Recognition and Image Analysis* 2011, **21(3), p.384**.
5. van Griethuysen JJM, Fedorov A, Parmar C, Hosny A, Aucoin N, Narayan V, Beets-Tan RGH, Fillion-Robin JC, Pieper S, Aerts H: **Computational Radiomics System to Decode the Radiographic Phenotype.** *Cancer Res* 2017, **77:**e104-e107.
